# DNA barcodes for 1/1000 of the animal kingdom

**DOI:** 10.1098/rsbl.2009.0848

**Published:** 2009-12-16

**Authors:** Paul D. N. Hebert, Jeremy R. deWaard, Jean-François Landry

**Affiliations:** 1 Biodiversity Institute of Ontario, University of Guelph, Guelph, Ontario, CanadaN1G 2W1; 2 Department of Forestry Science, University of British Columbia, Vancouver, British Columbia, CanadaV6T 1Z4; 3 Entomology, Royal British Columbia Museum, Victoria, British Columbia, CanadaV8W 9W2; 4 Research Centre, Agriculture and Agri-Food Canada, Ottawa, Ontario, CanadaK1A 0C6

**Keywords:** DNA barcoding, cytochrome *c* oxidase 1, species identification, cryptic species, Lepidoptera

## Abstract

This study reports DNA barcodes for more than 1300 Lepidoptera species from the eastern half of North America, establishing that 99.3 per cent of these species possess diagnostic barcode sequences. Intraspecific divergences averaged just 0.43 per cent among this assemblage, but most values were lower. The mean was elevated by deep barcode divergences (greater than 2%) in 5.1 per cent of the species, often involving the sympatric occurrence of two barcode clusters. A few of these cases have been analysed in detail, revealing species overlooked by the current taxonomic system. This study also provided a large-scale test of the extent of regional divergence in barcode sequences, indicating that geographical differentiation in the Lepidoptera of eastern North America is small, even when comparisons involve populations as much as 2800 km apart. The present results affirm that a highly effective system for the identification of Lepidoptera in this region can be built with few records per species because of the limited intra-specific variation. As most terrestrial and marine taxa are likely to possess a similar pattern of population structure, an effective DNA-based identification system can be developed with modest effort.

## 1. Introduction

The need for an advance in our ability to identify and discriminate species is widely acknowledged (Sutherland of Houndswood 2008). Most eukaryotes remain undescribed and the entry of a species into the Linnaean system does little to ensure its subsequent recognition because the subtle morphological characters that separate closely allied species often demand expert interpretation. Sequence diversity, in short, standardized gene regions (DNA barcodes), provides an alternative approach for both the identification of known species and the discovery of new ones (Hebert *et al*. 2003; Savolainen *et al*. 2005; Mitchell 2008). However, questions persist concerning the efficacy of DNA barcoding (Hickerson *et al*. 2006) and the level of effort that is required to parameterize an effective identification system (Zhang *et al*. in press).

## 2. Material and methods

We tested the ability of DNA barcodes to both identify known species and reveal overlooked taxa within the Lepidoptera of North America. With nearly 13 000 species, this assemblage includes 1 per cent of all described animal species. We acquired DNA barcodes from 11 289 individuals representing 1327 species (electronic supplementary material, figure S1) collected from the eastern half of this continent (38–59°N, 70–90°W). These taxa included representatives of 62 different families of micro- and macro-Lepidoptera (electronic supplementary material, figure S2), but with stronger representation for macros because of the greater maturity of their taxonomy. PCR amplification using a single pair of primers consistently recovered the 648 bp region near the 5′ terminus of the mitochondrial cytochrome *c* oxidase I (COI) gene that serves as the barcode region for the animal kingdom (Hebert *et al*. 2003). DNA isolation, PCR amplification and DNA sequencing followed standard protocols (deWaard *et al*. 2008). COI pseudogenes (NUMTS) have been encountered in some invertebrate lineages (e.g. Buhay 2009), but we detected none in our work, a result that coincides with their rarity in taxa with small genome sizes. Sequences were deposited in GenBank with accession codes GU087155–GU097197. Complete specimen data are available from the Barcode of Life Data System (www.barcodinglife.org) in the project ‘Lepidoptera of Eastern North America Phase I’.

## 3. Results and discussion

**Figure 1.**
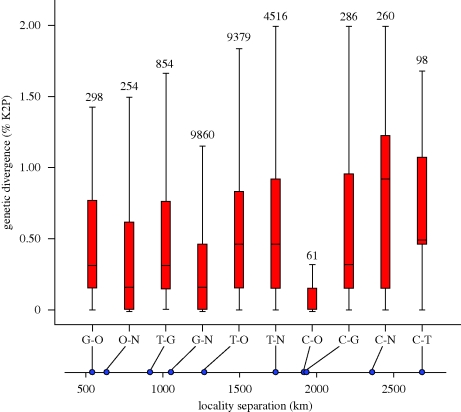
Box plots of intraspecific divergence observed for populations of species that were collected from two or more localities. The median distances of separation are given for the general localities of Churchill, MB (C), Guelph, ON (G), Ottawa, ON (O), St Andrew's, NB (N) and Great Smoky Mountains National Park, TN/NC (T). The number of comparisons used to calculate genetic divergence is denoted above each box plot.

Sequence analysis of the COI amplicon (electronic supplementary material, figure S2) established that members of a species usually showed low sequence variation, averaging 0.43 per cent (s.e. = 0.017%) while congeneric species possessed 18-fold higher mean divergences (7.70%, s.e. = 0.033%). The present study provides the first comprehensive analysis of barcode divergences in populations of single species separated by large geographical distances. Comparison of intraspecific divergences for populations collected from 500–2800 km apart revealed no significant increase in genetic distances with geographical separation (figure 1). This lack of substantial regional variation in barcode sequences indicates that an effective identification system can be constructed for the Lepidoptera fauna of eastern North America without extensive geographical surveys of each species. We anticipate that similarly muted levels of intraspecific variation will be shared by most taxa in other insect orders such as Coleoptera, Diptera and Hymenoptera from this region. We expect more differentiation in groups with low vagility and in other areas, such as western North America, where higher topographic roughness provides more opportunities for population isolation and differentiation. It will also be intriguing to probe the patterns of regional divergence in areas such as Australia where Pleistocene glaciations had a much less dramatic impact on species distributions.

We detected only nine cases of barcode sharing in the 1327 species included in our study, all involving situations in which a pair of species shared the same barcode. These cases always involved congeneric species with close morphological similarity. Because 99.3 per cent of the 1327 species had barcode sequences distinct from those of other taxa, a COI reference library can generate identifications very effectively.

**Figure 2.**
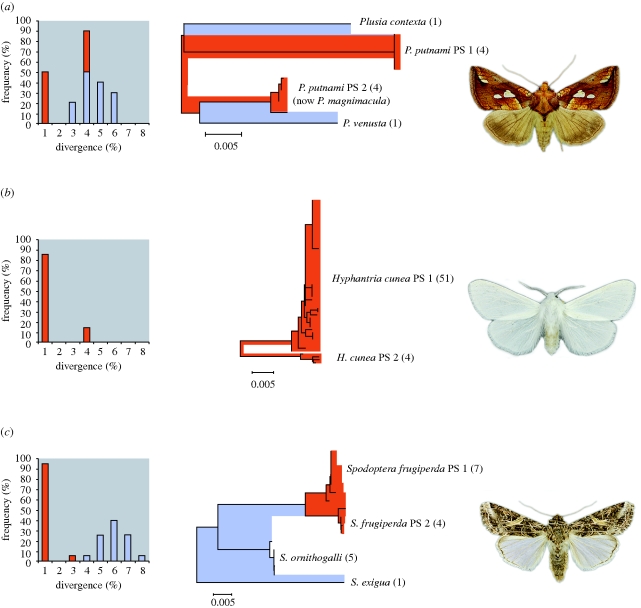
Histograms and neighbour-joining trees showing deep sequence divergences at COI among species in three genera of Lepidoptera from eastern North America: (*a*) *Plusia*, (*b*) *Hyphantria* and (c) *Spodoptera*. For the species displaying deep divergences, intraspecific divergences are shaded red and divergences among congeneric taxa are shaded blue. Individuals showing deep ‘intraspecific’ barcode divergence occur in sympatry for all three taxa.

Although most species possessed low intra-specific divergence, 67 taxa included two or three barcode groups with more than 2 per cent sequence divergence. Many of these cases probably reflect overlooked species pairs or triads. As evidence, we note that individuals of *Plusia putnami* separated into two barcode groups with 3.8 per cent COI divergence (figure 2*a*). Subsequent investigation revealed differences in genitalia, host plant use and habitats, leading to the description of a new species (Handfield & Handfield 2006). Other cases of deep barcode divergence involved species where there is independent evidence for unrecognized taxa. For example, two barcode lineages with 2.8 per cent sequence divergence were detected in the fall webworm, *Hyphantria cunea* (figure 2*b*), which has long been thought to include two species with differing larval morphologies (Itô & Warren 1973). Young species pairs will be overlooked by a 2 per cent screening threshold, but they can still show barcode differentiation. For example, the fall armyworm, *Spodoptera frugiperda*, includes two barcode lineages with 1.3 per cent divergence (figure 2*c*). This species consists of two ‘host races’ that not only have different primary hosts (rice versus corn), but show allozyme and mitochondrial DNA divergence as well as reproductive isolation (Levy *et al*. 2002), justifying their recognition as distinct species. As this last example reveals, barcodes can highlight young species pairs, but studies of biological covariates are critical to confirm their status.

Our work affirms the validity of most Lepidoptera species recognized though prior taxonomy and suggests that relatively few species have been overlooked as just 5.1 per cent of the 1327 taxa included deeply divergent barcode lineages. However, there are two provisos. Young species pairs, such as those comprising *S. frugiperda*, will often be morphologically cryptic, and will also show low barcode divergence. Such taxa can be revealed, but only through a search for covariation between barcode splits and ecological or morphological traits. Secondly, the constrained species discovery in this study probably reflects both the intensity of prior taxonomic work on Lepidoptera and their flamboyant phenotypes. Interestingly, the incidence of overlooked species encountered in the present study shows close congruence to the value reported for a well-studied fauna of tropical Lepidoptera (Hajibabaei *et al*. 2006). In contrast, barcode analyses on insect groups with cryptic morphologies have encountered much higher rates of species discovery (Smith *et al*. 2006, 2008).

In summary, this study has assembled DNA barcodes for 0.1 per cent of the animal species described over the past 250 years. Our results confirm the effectiveness of a DNA barcode reference library in the identification of a continental fauna of Lepidoptera, reinforcing conclusions from studies that examined fewer species and that probed diversity on smaller geographical scales. Our work has also provided further examples of deep barcode divergences, illuminating probable overlooked species, and setting the stage for their detailed taxonomic investigation. There is no reason to expect that Lepidoptera are a particularly compliant target for barcode-based identification systems. Instead, it is likely that the key findings of this investigation apply to most other taxonomic groups occupying continental or oceanic habitats. In such situations, the barcode analysis of very few individuals of each species will provide the basis for a highly effective identification system. More effort will be required to gain a good understanding of sequence diversity in taxa from insular or freshwater habitats where local population differentiation is more pronounced, but such taxa form a minor component of global biodiversity.

We conclude that DNA barcoding can deliver—in its promise both to enable the automated identification of known species and to aid the detection of overlooked taxa. Further, as this study indicates, a comprehensive barcode library for animal life can be assembled rapidly, promising massive improvement in our knowledge of biodiversity.
